# Long-Term Results (up to 20 Years) of 19 mm or Smaller Prostheses in the Aortic Position. Does Size Matter? A Propensity-Matched Survival Analysis

**DOI:** 10.3390/jcm10102055

**Published:** 2021-05-11

**Authors:** Horea Feier, Andrei Grigorescu, Lucian Falnita, Oana Rachita, Marian Gaspar, Constantin T. Luca

**Affiliations:** 1 Department of Cardiology, University of Medicine and Pharmacy, 300391 Timisoara, Romania; ae.grigorescu@gmail.com (A.G.); mariangaspar24@yahoo.ro (M.G.); costiluca67@yahoo.ro (C.T.L.); 2 Divisions of Cardiovascular Surgery and Cardiology, Institute for Cardiovascular Diseases, 300391 Timisoara, Romania; lfalnita@gmail.com (L.F.); droanarachita@gmail.com (O.R.)

**Keywords:** aortic valve, small root, mismatch, 19 mm, PPM

## Abstract

Background: The long-term performance of prostheses in the small aortic root is still unclear. Methods: Patients who received a 21 mm or smaller aortic valve between 2000–2018 were retrospectively analyzed. Propensity matching was used in order to account for baseline differences in 19 mm vs. 21 mm valve subgroups. Results: Survival at 10 years was 55.87 ± 5.54% for 19 mm valves vs. 57.17 ± 2.82% for 21 mm ones in the original cohort (*p* = 0.37), and 58.69 ± 5.61% in 19 mm valve recipients vs. 53.60 ± 5.66% for 21 mm valve subgroups in the matched cohort (*p* = 0.55). Smaller valves exhibited significantly more patient–prothesis mismatch (PPM) than larger ones (87.30% vs. 57.94%, *p* < 0.01). All-cause mortality was affected by PPM at 10 years (52.66 ± 3.28% vs. 64.38 ± 3.87%, *p* = 0.04) in the unmatched population. This difference disappeared, however, after matching: survival at 10 years was 51.82 ± 5.26% in patients with PPM and 63.12 ± 6.43% in patients without PPM. (*p* = 0.14) Conclusions: There is no survival penalty in using 19 mm prostheses in the small aortic root in the current era. Although PPM is more prevalent in smaller sized valve recipients, this does not translate into reduced survival at 10 years of follow-up.

## 1. Introduction

Aortic stenosis is currently the most frequent valve disease [1]. The prevalence of the disease increases with age, reaching 3.9% in those aged over 70 years and 9.8% in people aged over 80 years [2]. Aortic valve replacement is recommended for symptomatic severe aortic valve stenosis, as well as asymptomatic patients with a reduced left ventricular ejection fraction (LVEF) or undergoing surgery for another indication [3,4]. The expanding indications for transcatheter aortic valve replacement (TAVR) are challenging the established therapeutic protocols for surgical aortic valve replacement (SAVR), particularly in patients with a high surgical risk. 

The small aortic root has been defined in the surgical series as an aortic annulus accepting an aortic prosthesis smaller than or equal to 21 mm in size [5,6]. In this population, surgeons have the choice of implanting a small stented prosthesis, a stentless one, a rapid-deployment valve, or performing aortic annulus enlargement, which can increase the operation time and the surgical risk. 

We sought to investigate the long-term results (up to 20 years) of very small prostheses (19 mm in size or smaller) in the aortic position.

## 2. Materials and Methods

### 2.1. Data Collection

This report represents a comparative retrospective single-center study. Patient data was collected during treatment using standardized forms to record demographic and clinical characteristics as well as procedural and follow up data. Follow-up was obtained using medical records, patient interviews and National Health Register. The study protocol was approved by the Ethical Board of our Institution. Due to the retrospective nature of the study, written consent was waived. 

### 2.2. Patient Population

We reviewed the hospital records of patients operated on in our department from 1 January 2000 to 31 December 2018. During this time frame, we performed 13,227 open-heart procedures in adult (>18 years) patients. We included in the study all patients that received a 21 mm or smaller valve in the aortic position, irrespective of the underlying pathology, and found 670 subjects that satisfied these criteria. While most patients underwent surgery for isolated aortic stenosis, associated procedures included coronary artery bypass, mitral valve, or ascending aortic replacements. 

Within this cohort, we compared the long-term results of patients who received a 19 mm or smaller valve substitute (*n* = 132) with those of a matched sample receiving a 21 mm prosthesis (*n* = 538). There were 5 patients who received an 18 mm valve (Medtronic Open Pivot AP18) and 127 patients received a 19 mm valve substitute.

### 2.3. Definitions

Patients with an aortic annulus who would accept a 19 mm valve or smaller were defined as having a small aortic annulus. The effective orifice area (EOA) of the protheses that were implanted were taken from manufacturers’ fact sheets, as previously reported and validated [7,8]. Subjects were classified as having patient–prosthesis mismatch (PPM) if the indexed EOAi ≤ 0.85 cm^2^/m^2^. Severe PPM was considered to be present if EOAi ≤ 0.65 cm^2^/m^2^. Very small prostheses were those ≤19 mm in size. Early mortality was death occurring within 30 days of operation, in-hospital or not. Severe pulmonary hypertension (PHT) was defined as a systolic pulmonary artery pressure superior to 55 mm Hg. Peripheral vascular disease (PVD) was defined as a >50% stenosis in the carotid, subclavian, or peripheral arteries. Coronary artery disease (CAD) was considered to be present when a major epicardial artery presented a stenosis >50%. Chronic obstructive pulmonary disease (COPD) was defined by spirometry as a forced expiratory volume (VEMS) lower than 80% than the normalized value, under inhalatory medication. 

### 2.4. Propensity Matching

**Figure 1:**
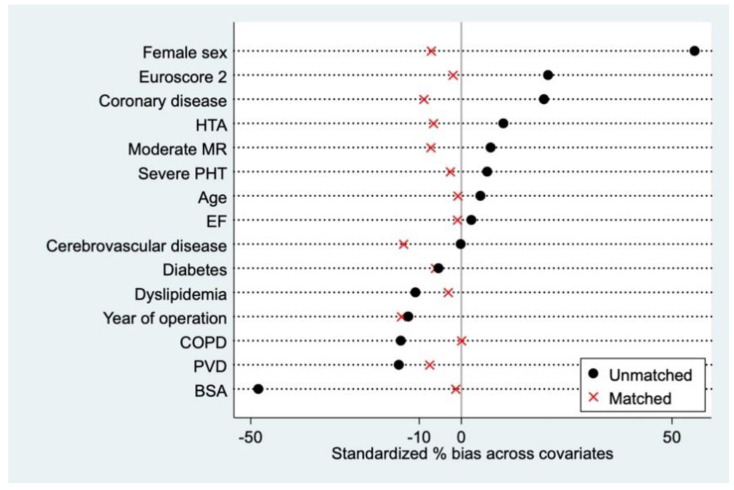
Bias before and after propensity matching.

Patients who received a 19 mm valve were matched 1:1 to those receiving a 21 mm prosthesis with a 0.2 standard deviation caliper by matching without replacement using the “psmatch2” statistical package in Stata. Matching covariates were the following: year of operation, age, sex, body surface area, EuroSCORE II surgical risk score, ejection fraction, severe pulmonary hypertension, residual ≥ grade 2 mitral regurgitation, coronary artery disease, dyslipidemia, arterial hypertension, chronic obstructive pulmonary dysfunction, diabetes, peripheral vascular disease, and cerebrovascular disease. Matching resulted in a 252-patient cohort. The average treatment on the treated is estimated from this sample. Covariate balance was assessed after matching by assessing the standardized percent bias, with less than 10% considered acceptable (Figure 1). 

The baseline characteristics of our patients, as well as the matched cohort, are presented in Table 1. 

### 2.5. Objectives

The primary endpoint was overall survival. Patients were confirmed as dead by telephone interview with their relatives and the exact date of death was found in the National Health Register. Follow-up was 100% complete. The secondary outcome of interest was a composite endpoint of death or reoperation for a prothesis-related issue. These were performed for major paravalvular leak, endocarditis, degeneration, prosthesis mismatch, or valve thrombosis. Patients who were subsequently reoperated for another issue, such as aortic surgery, coronary artery bypass, or mitral valve, were not censored in the survival analysis. 

### 2.6. Statistical Analysis

Normal distribution was assessed using the Shapiro–Wilk test. Univariate analysis was performed using *t*-tests, Mann–Whitney or chi-squared tests. Time-to-event analysis was performed using log-rank or Kaplan–Meier estimates. Cox proportional hazard regression was performed for the primary and secondary outcome, with data reported as odd ratios and 95% confidence intervals. Multivariate survival analysis was performed on the unmatched population using covariates and on the matched population using valve size or patient–prosthesis mismatch as the only covariate. The proportional hazard assumptions were tested using Schoenfeld residuals analysis. Linearized rates of death or reoperation were calculated as being the incidence of the event divided by total patient-years of follow-up and reported for both the unmatched and matched populations. Statistical testing was performed using Stata 16.1 (StataCorp LLC, College Station, TX, USA). A *p* value less than 0.05 was deemed to assess statistical significance. 

## 3. Results

Among 670 unmatched patients, there were 132 aortic valve replacements with prostheses size 19 or smaller, while 538 received a 21 mm prosthesis. There were 4 patients with an 18 mm prosthesis and 128 with 19 mm valves. Mechanical prostheses were implanted in 414 patients and 256 patients underwent aortic valve replacement with xenografts (Table 2).

Matching yielded two groups of 126 patients that received either a 19 mm and smaller valve replacement, or a 21 mm prosthesis (Table 1). There were no biologic valves with size 19 or smaller available in our unit prior to 2008. Thus, we implanted a mechanical prosthesis prior to this date whenever we encountered an extremely small aortic annulus, even in patients > 65 years. Associated procedures were performed in 19 (15.08%) patients with a small aortic annulus, vs. 15 (11.90%) patients with larger ones (*p* = 0.46). These procedures included two patients with annulus enlargement (and a subsequent 19 mm valve insertion), as well as two patients with ascending aortic replacement (Table 3). Patients with a small aortic annulus had slightly longer cross-clamp and bypass times. Fourteen patients had a postoperative length-of-stay >15 days. Of these, two received a 21 mm prosthesis associated with mitral valve repair and ascending aortic replacement, respectively. Eleven patients who required a longer postoperative stay had a 19 mm valve implanted. One of these had a 19 mm valve implanted after aortic annulus enlargement. She had a complicated postoperative course, with an intra-aortic balloon pump placement, low output syndrome, renal failure requiring dialysis, and several episodes of sepsis. She was never discharged and died after 277 days.

Early mortality (<30 days) was 4.76% and was not different between patients with a small aortic annulus or not (*p* = 0.55). The predicted mortality by the EuroSCORE II risk score of these patients was 2.54 ± 3.07 and 2.59 ± 2.10, respectively. 

**Figure 2:**
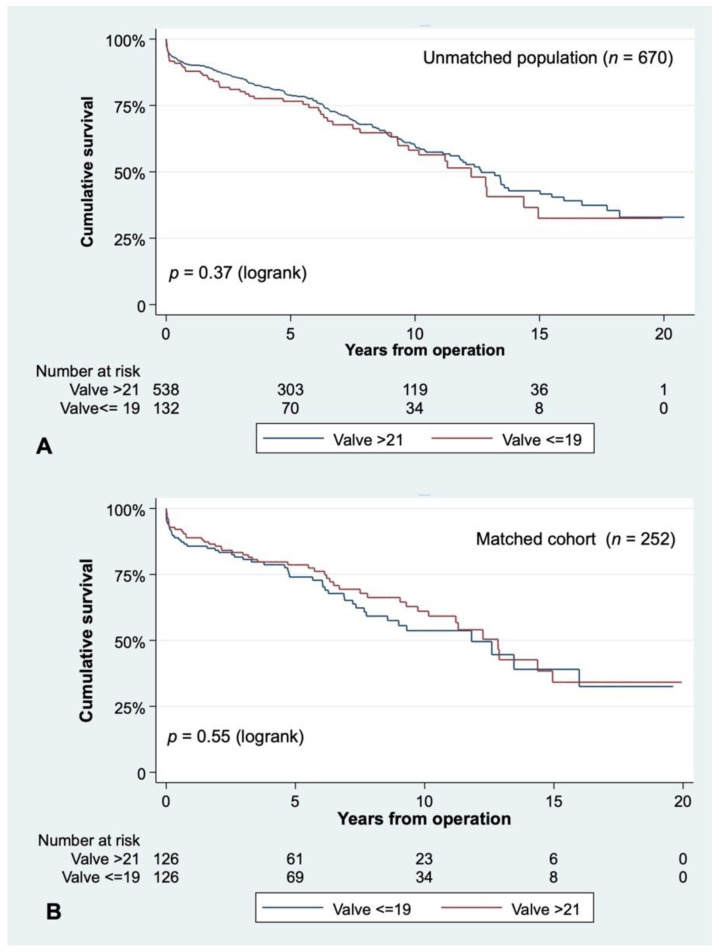
Cumulative survival for the unmatched (**A**) and matched cohorts (**B**), according to valve size.

The unmatched cohort included 4093 patient-years of follow-up (the mean follow-up was 6.62 ± 4.61 years), whereas the matched one had a mean follow-up of 6.26 ± 4.53. Ten-year survival of the unmatched population was 55.87 ± 5.54% for patients with an aortic annulus ≤ 19 mm and 57.17 ± 2.82% for patients with larger ones (*p* = 0.37, Figure 2A). 

**Figure 3:**
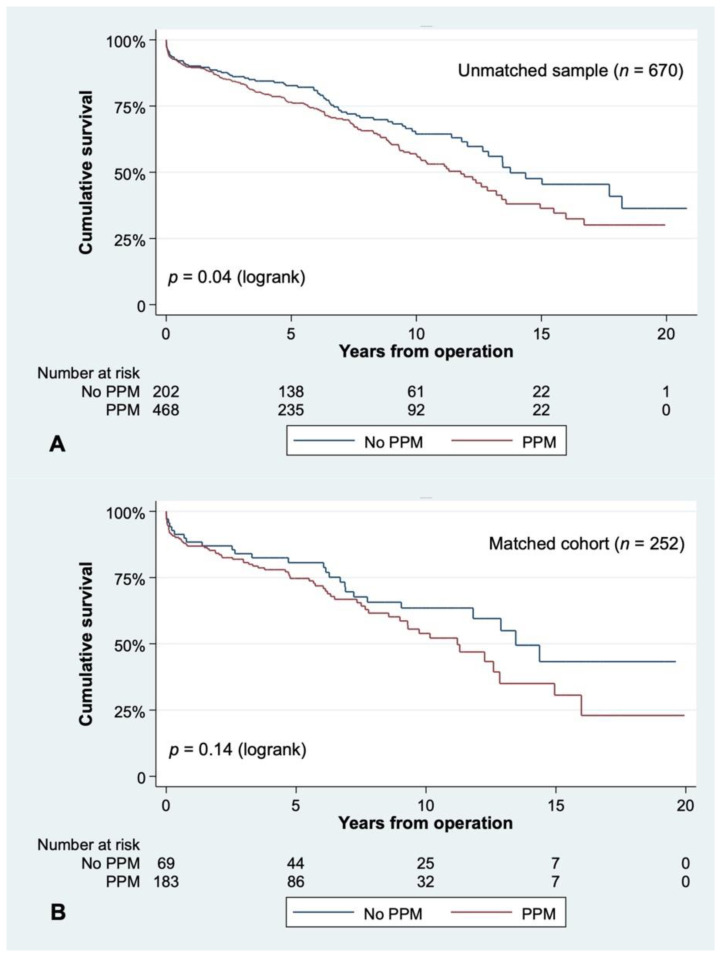
Long-term survival according to the presence of PPM (EOAi ≤ 0.85 cm^2^/m^2^) in the unmatched (**A**) or matched (**B**) sample. When matching for risk factors, there was no difference in survival.

**Figure 4:**
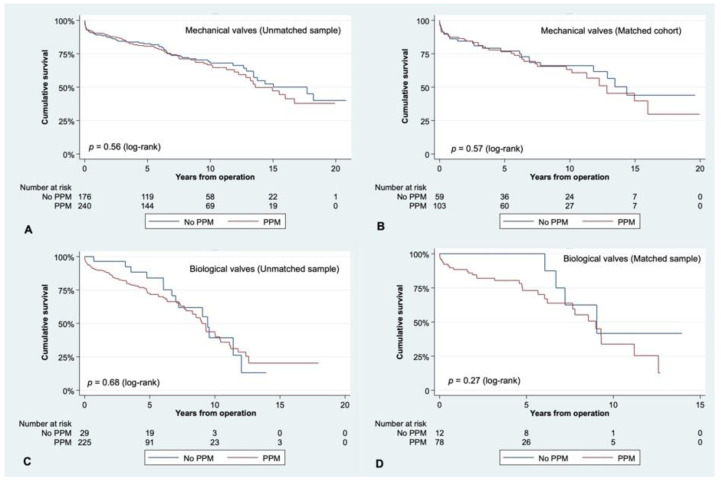
Long-term survival according to the presence of PPM (EOAi ≤ 0.85 cm^2^/m^2^)for mechanical valves recipients in the unmatched (**A**) or matched sample (**B**), as well as biologic valve patients in the unmatched (**C**) or propensity-matched sample (**D**).

When matching for preoperative variables, patients with the smallest prostheses still had similar survival as patients who received larger ones: survival at 10 years was 58.69 ± 5.61% for those receiving a 19 mm valve or smaller vs. 53.60 ± 5.66% for those with a 21 mm aortic substitute (*p* = 0.55, Figure 2B). The indexed EOA of very small prostheses (≤19 mm) was significantly lower than the one from 21 mm ones, either in the unmatched cohort (0.67 ± 0.14 vs. 0.81 ± 0.18, *p* < 0.01) or in the matched population (Table 2). Smaller valves exhibited significantly more PPM than larger ones (87.30% vs. 57.94%, *p* < 0.01). All-cause mortality was affected by PPM at 10 years (52.66 ± 3.28% vs. 64.38 ± 3.87%, *p* = 0.04) after the primary operation in the unmatched population (Figure 3A). However, in the matched sample, survival at 10 years stood at 51.82 ± 5.26% in patients with PPM as opposed to 63.12 ± 6.43% in patients without PPM, respectively (*p* = 0.14) (Figure 3B). When looking at the influence of PPM in valve-type subgroups, we found it did not influence long-term survival in the unmatched or matched population (Figure 4). 

Independent risk factors for mortality within the unmatched population included older age, longer bypass times, serum creatinine, ejection fraction, diabetes, and peripheral vascular disease. In the matched population, neither valve size nor the presence of PPM would influence long-term survival (Table 4). Linearized rate of death was 0.05% per patient-year for the unmatched population and 0.06% per patient-year in the matched population.

There were eight reoperations in our unmatched sample for valve-related issues. The linearized rate of reoperation was 0.06% per patient-year overall. Reasons for reoperation were valve degeneration (3), PPM (2), endocarditis (2) or major paravalvular leak (1). There were no instances of valve thrombosis requiring thrombolysis or reoperation. Patients with major paravalvular leak or prosthetic valve endocarditis were reoperated within 1 year after the initial operation, while those with biologic valve degeneration or PPM were reoperated after a mean of 7.4 years. Two of those reoperations were in 19 mm valve recipients (for degeneration and PPM), while the others were in patients with 21 mm valves. The secondary end-point of survival without death or reoperation was met at 10 years by 54.35 ± 5.60% of patients receiving a 19 mm or smaller prosthesis and by 56 ± 2.82% of those with a 21 mm valve in the unmatched sample (*p* = 0.33), while this was true for 57.1 ± 5.69% and 51.37 ± 5.70% in the matched population (*p* = 0.57). 

## 4. Discussion

Aortic stenosis is the most frequent valve pathology. Its incidence has increased due to the longer life expectancy afforded by advances in healthcare. While aortic valve treatment has been traditionally reserved for open surgery, the development of TAVR has challenged this [9,10,11]. Surgeons tend to strive to implant a prosthesis as big as possible, with the goal of delivering a low-gradient, high EOA valve, that would mimic, as close as possible, a healthy, normal, functioning aortic valve. This presents a problem in patients with a very small aortic annulus (≤19 mm), in which a small sized, stented prosthesis would inherently lead to higher postoperative gradients. Stentless valves show significantly lower gradients and higher EOA’s than stented prostheses at mid-term follow-up [12], but survival was similar. Another option would be to perform aortic root enlargement [13,14,15]. Several techniques have been described [16,17] and they do not increase surgical risk in experienced hands [18]. Finally, rapid-deployment valves have been shown to perform well in the small aortic root [19,20] and they are a valid alternative to root enlargement in this population. However, the key question remains: do we need to perform aortic root enlargement or to employ low-gradient prostheses in the small aortic root?

There are currently limited data regarding the long-term use of very small prostheses (≤19 mm) in the aortic position. Most 19 mm valve implants have at least moderate PPM (>0.65 cm^2^/m^2^, <0.85 cm^2^/m^2^), so the issue of long-term survival is closely linked to the issue of patient–prosthesis mismatch for this valve size. Rahimtoola was the first to introduce the notion of PPM into clinical practice [21]. While some authors have shown PPM to be responsible for reduced early and late survival following SAVR [7,22,23,24], others have failed to do so [25,26], and functional recovery after SAVR does not seem to be affected by PPM [27]. In a small series, several papers have reported excellent long-term results with 19 mm and smaller stented prostheses with no survival penalty [25,28,29,30,31]. Is the conviction that bigger is always better really justified?

This is one of the biggest studies on 19 mm valves. Our initial population subgroups were unbalanced, as there were more women, BSA and BMI were smaller, coronary disease was more prevalent, and surgical risk was greater in patients receiving a 19 mm valve. By matching for these factors, as well as others (such as year of surgery, age, cardiac status, and comorbidities), we obtained two balanced groups. We could not find differences in long-term survival in patients who received a 19 mm prosthesis compared to those who had a 21 mm implanted, in the unmatched or matched population. With regard to PPM, there was a significant survival difference at 10 years in the original cohort in patients with moderate to severe PPM (EOAi ≤ 0.85 cm^2^/m^2^). Nevertheless, when matching the two cohorts, these differences disappeared, suggesting that other factors were responsible for the impaired survival. 

Our results are in line with other reports that found no survival difference in isolated aortic valve replacements with 19 mm or 21 mm prostheses up to 30 years after the primary procedure, even in patients with PPM [32]. Others authors that have studied modern prostheses have found that PPM did not adversely affect long-term survival and that valve size may be irrelevant [33,34,35]. 

In the case of patients with aortic stenosis, there is the widely accepted view that clinical improvement is due to the reduction of transvalvular gradients. Persistently elevated transvalvular gradients impact left ventricular hypertrophy and mass regression after SAVR [36]. Stented valves are inherently stenotic due to the presence of the sewing ring and of the prosthetic valve stents, but PPM is virtually nonexistent with the latest generation of tissue valves, including prostheses sized 19 mm or smaller [30,37]. So, is there still a rationale behind proposing an aortic annulus enlargement? In a recent multi-institutional study, Tam and associates report that root enlargement does not increase surgical risk but it fails to produce a survival advantage at 8 years after SAVR [15]. The interest of this procedure at the present time could be that it would allow an easier valve-in-valve implantation by TAVR in the event of late bioprosthetic degenerescence. Indeed, although the feasibility of a valve-in-valve in 19 mm degenerated bioprostheses implantation has been documented [38], the procedure requires the fracturing of the bioprosthetic ring by aggressive dilatation [39], and the results are suboptimal [40,41].

### Study Limitations

The main limitation of the present study is that follow-up echocardiographic data were taken at different time points by different examiners and, as such, could not be used. If recent ultrasound data had been available, ideally performed by the same examiner, it would have allowed actual in-vivo assessment of EOA and other indices, such as energy-loss index [42], that might better appreciate the flow impedance in valve recipients. Another limitation is the inability to draw definitive conclusions about the influence of PPM on outcomes beyond 10 years. In our sample, there was a trend for patients with PPM to fare worse 10 years after the primary procedure but to investigate this would require a longer follow-up. 

## 5. Conclusions

The use of modern 19 mm aortic prostheses does not carry a survival penalty in patients up to 10 years of follow-up. Moderate to severe patient–prosthesis mismatch did not influence long-term results when matching for other covariates. If present, differences in survival might appear only beyond 10 years. As expected, age, cardiopulmonary bypass times, ejection fraction, diabetes, peripheral vascular disease, and renal dysfunction are independent risk factors for late death in the unmatched population. Surgeons should not fear the use of small aortic prostheses as they are not associated with worse early or late results in the current era.

## Figures and Tables

**Table 1:** Baseline characteristics of the study cohort.

Variable	All Patients			Matched Cohort		
	Valve ≤ 19 (*n* = 132)	Valve ≥ 21 (*n* = 538)	*p* Value	Valve ≤ 19 (*n* = 126)	Valve ≥ 21 (*n* = 126)	*p* Value
	*n* (%)	*n* (%)		*n* (%)	*n* (%)	
Age	64.55 ± 11.08	64.82 ± 12.45	0.54	64.39 ± 11.20	64.5 ± 13.95	0.94
Female sex	109 (82.59)	311 (57.81)	<0.01	103 (81.75)	107 (84.92)	0.49
EuroSCORE II	2.67 ± 3.16	2.11 ± 1.80	<0.01	2.54 ± 3.07	2.59 ± 2.10	0.87
Aortic stenosis	121 (91.67)	510 (94.80)	0.16	115 (91.27)	116 (92.06)	0.82
Arterial hypertension	89 (67.42)	340 (63.20)	0.36	84 (66.67)	88 (69.84)	0.58
Severe pulmonary hypertension	14 (10.61)	46 (8.55)	0.45	14 (11.11)	15 (11.90)	0.84
Diabetes	22 (16.67)	102 (18.96)	0.54	20 (15.87)	23 (18.25)	0.61
CAD ^1^	24 (18.18)	61 (11.34)	0.03	20 (15.87)	24 (19.05)	0.5
COPD ^2^	2 (1.52)	22 (4.09)	0.15	2 (1.59)	2 (1.59)	1.00
Dyslipidemia	61 (46.21)	282 (52.42)	0.20	60 (47.62)	62 (49.21)	0.80
PVD ^3^	4 (3.03)	33 (6.13)	0.16	4 (3.17)	6 (4.76)	0.51
Cerebrovascular disease	4 (3.03)	18 (3.35)	0.85	3 (2.38)	6 (4.76)	0.30
LV-EF ^4^	52.41 ± 9.19	52 ± 9.60	0.66	52.82 ± 8.62	52.92 ± 10.30	0.93
Moderate mitral regurgitation	35 (26.32)	65 (21.74)	0.29	33 (26.19)	37 (29.37)	0.57
Moderate tricuspid regurgitation	9 (6.82)	21 (3.90)	0.14	9 (7.14)	7 (5.56)	0.60
BSA ^5^	1.71 ± 0.19	1.80 ± 0.19	<0.01	1.71 ± 0.19	1.71 ± 0.20	0.91
Mean aortic gradient	55.78 ± 21.06	54.14 ± 19.01	0.39	55.61 ± 21.46	52.72 ± 18.91	0.26
BMI ^6^	26.05 ± 4.62	26.97 ± 4.89	0.04	26.06 ± 4.51	26.15 ± 5.33	0.88

^1^ CAD: a stenosis of at least 50% in a major epicardic coronary vessel; ^2^ COPD: chronic pulmonary obstructive disease diagnosed by spirometry and/or under inhalatory medication; ^3^ PVD: peripheral vascular disease or carotid stenosis > 50%; ^4^ LV-EF: left ventricular ejection fraction; ^5^ BSA: body surface area measured by Mosteller’s formula; ^6^ BMI: body mass index.

**Table 2:** Prostheses used in our sample.

Valve Type	All Patients		Matched Cohort	
	*n*	%	*n*	%
Mechanic				
Sorin Carbomedics TopHat	121	18.05	43	17.06
St Jude Masters	113	16.86	45	17.86
Carbomedics Orbis Model 100	43	6.41	19	7.54
Medtronic Open Pivot	37	5.52	15	5.95
Sorin Bicarbon	34	5.07	16	6.35
St Jude Regent	30	4.48	9	3.57
Carbomedics Standard	15	2.24	9	3.57
Medtronic Hall	11	1.64	2	0.79
Sorin Allcarbon	5	0.75	3	1.19
Medtronic Advantage	4	0.6	0	0
ON-X	2	0.3	0	0
St Jude HP	1	0.15	1	0.4
Biologic				
Edwards Perimount 2900	113	16.87	41	16.27
Medtronic Hancock II	74	11.05	22	8.73
St Jude Epic	29	4.33	13	5.16
Braile FABP	17	2.54	7	2.78
Sorin Mitroflow	7	1.04	1	0.4
Medtronic Freestyle	6	0.9	4	1.59
Sulzer Carbomedics Labcor	5	0.75	1	0.4
St Jude Trifecta	2	0.3	1	0.4
Sorin Pericarbon	1	0.15	0	0
Total	670	100	252	100

**Table 3:** Intraoperative data, in-hospital, and long-term outcomes of matched cohort.

Variable	Valve ≤ 19 *n* (%)	Valve ≥ 21 *n* (%)	*p*
Concomitant procedures	15.08%	11.9%	0.46
CABG	9 (7.14)	8 (6.35)	0.80
Mitral valve replacement	7 (5.56)	2 (1.59)	0.09
Mitral valve repair	1 (0.79)	3 (2.59)	0.27
Aortic annulus enlargement	2 (1.58)	1 (0.79)	0.56
Ascending aortic replacement	0 (0)	1 (0.79)	0.31
Carotid endarterectomy	1 (0.85)	0 (0)	0.33
Operative data			
Bypass time (mean ± SD)	120.23 ± 88.17	102.73 ± 38.35	0.04
Cross-clamp time (mean ± SD)	77.73 ± 43.43	66.72 ± 16.71	<0.01
Postoperative length of stay (mean ± SD)	12.08 ± 25.20	9.22 ± 4.79	0.21
Early mortality (<30 days)	5 (3.97)	7 (5.56)	0.55
EOAi (cm^2^/m^2^)	0.67 ± 0.13	0.84 ± 0.20	<0.01
PPM	110 (87.30)	73 (57.94)	<0.01
Severe PPM	68 (53.97)	18 (14.29)	<0.01
EuroSCORE II risk score	2.54 ± 3.07	2.59 ± 2.10	0.87
Follow-up (years) (mean ± SD)	6.54 ± 4.60	5.98 ± 4.47	0.32

**Table 4:** Cox proportional hazard regression of the all-cause mortality in the unmatched population (*n* = 670) and in the matched population, with valve size or PPM as risk factors in the propensity-matched cohort (*n* = 252).

Variable	All Patients (*n* = 670)		Matched Cohort (*n* = 252)	
	HR (95% CI)	*p* Value	HR (95% CI)	*p* Value
Valve size	0.92 (0.62–1.35)	0.68	0.76 (0.49–1.17)	0.22
PPM	1.06 (0.76–1.48)	0.72	1.56 (0.95–2.54)	0.07
Male sex	0.84 (0.62–1.16)	0.30		
Age (per year)	1.04 (1.03–1.06)	<0.01		
Arterial hypertension	0.92 (0.67–1.28)	0.65		
Bypass time (per min)	1.005 (1.003–1.008)	<0.01		
Dyslipidemia	0.77 (0.56–1.06)	0.11		
Creatinine (per mg%)	1.30 (1.13–1.50)	<0.01		
CAD ^1^	1.25 (0.83–1.88)	0.27		
Ejection fraction	0.98 (0.97–0.99)	0.04		
COPD ^2^	0.73 (0.31–1.70)	0.47		
Diabetes	1.63 (1.14–2.52)	<0.01		
BMI	0.97 (0.99–1.01)	0.17		
PVD ^3^	1.94 (1.16–3.25)	0.01		
Cerebrovascular disease	1.70 (0.82–3.53)	0.14		

^1^ CAD: a stenosis of at least 50% in a major epicardic coronary vessel; ^2^ COPD: chronic pulmonary obstructive disease diagnosed by spirometry and/or under inhalatory medication; ^3^ PVD: peripheral vascular disease or carotid stenosis > 50%.
